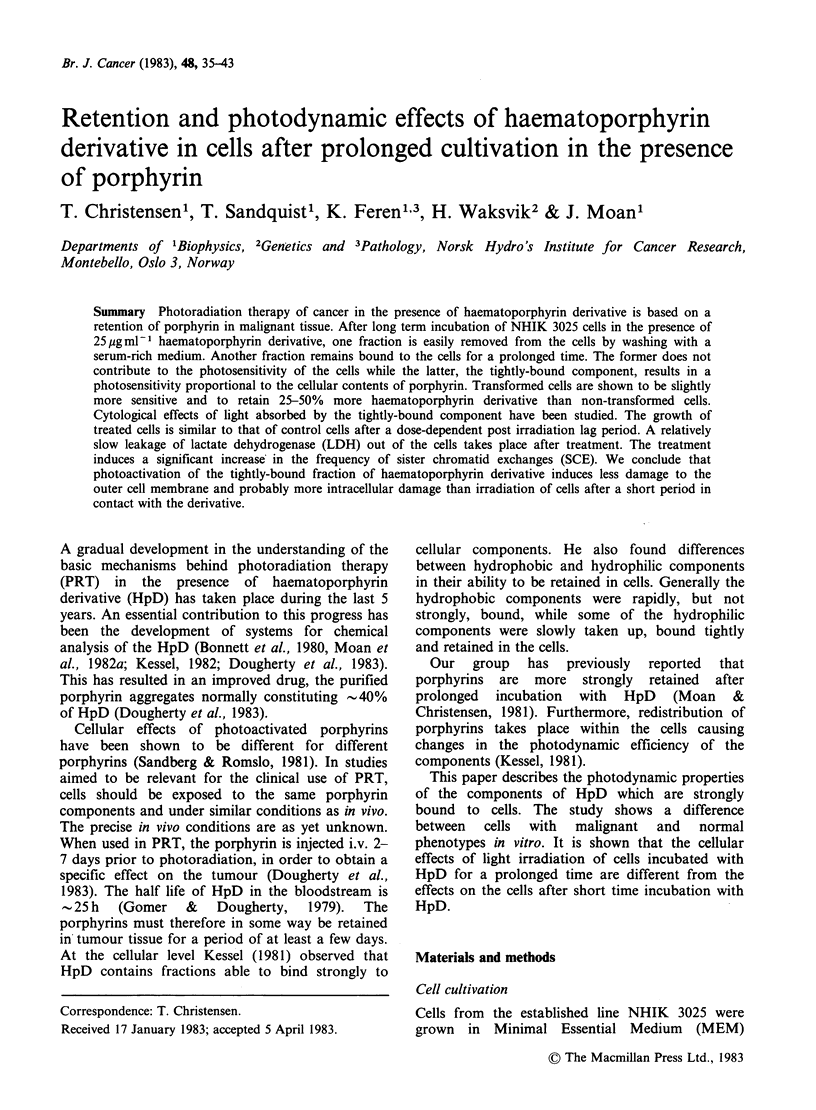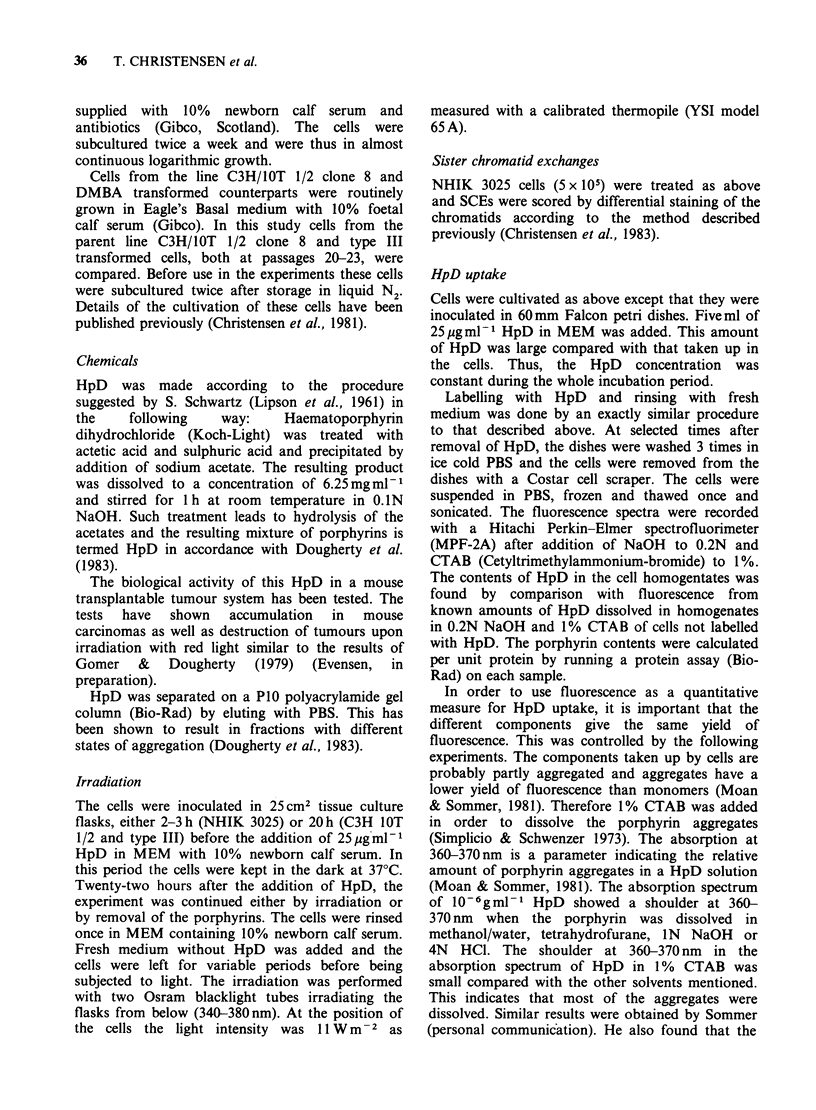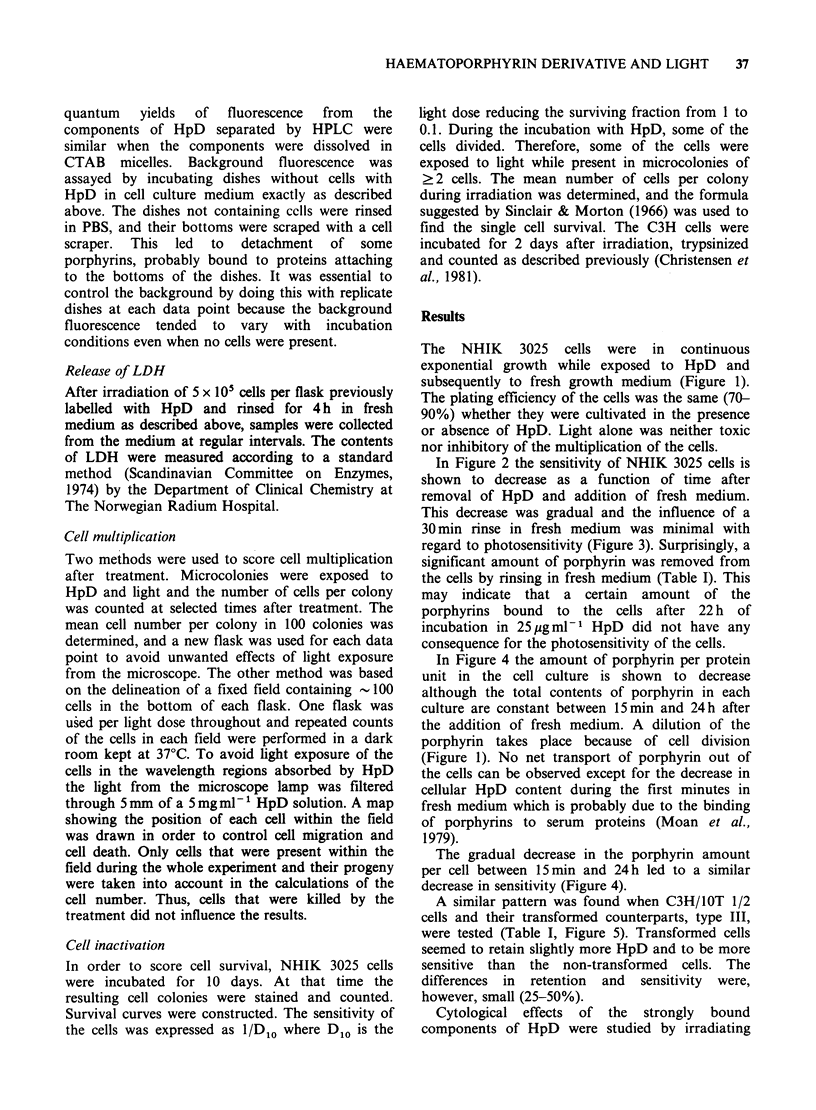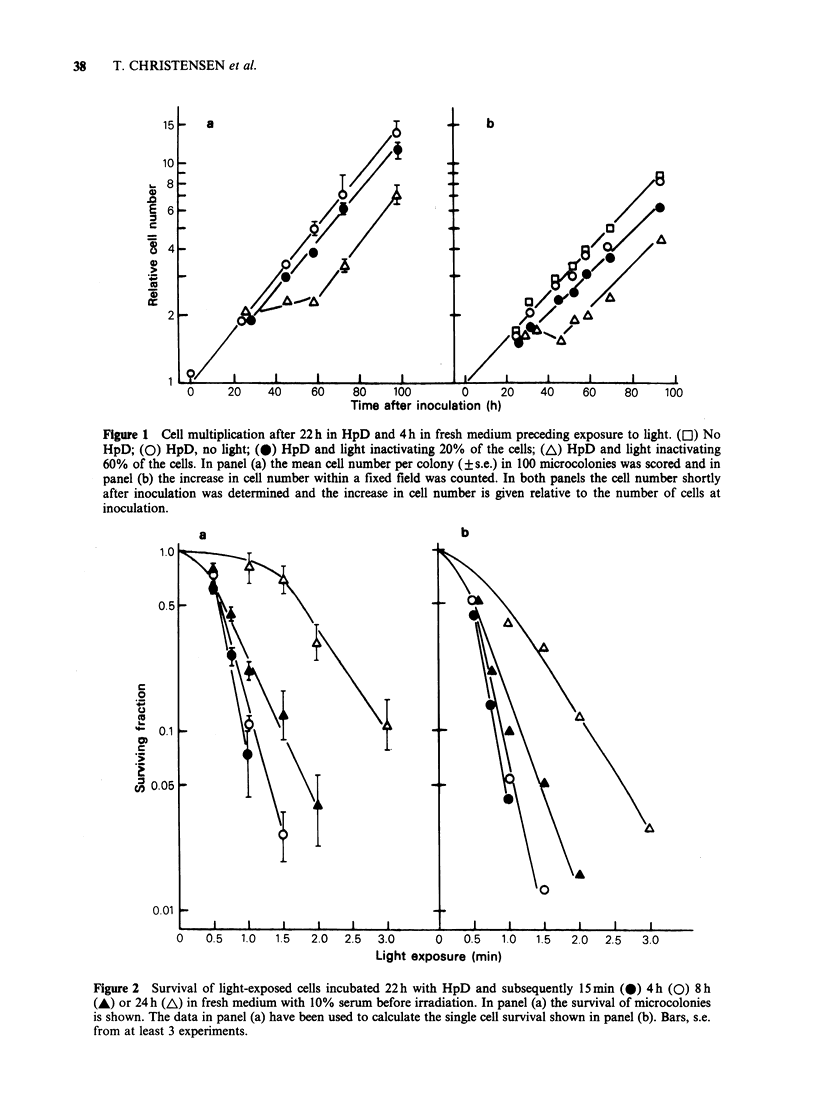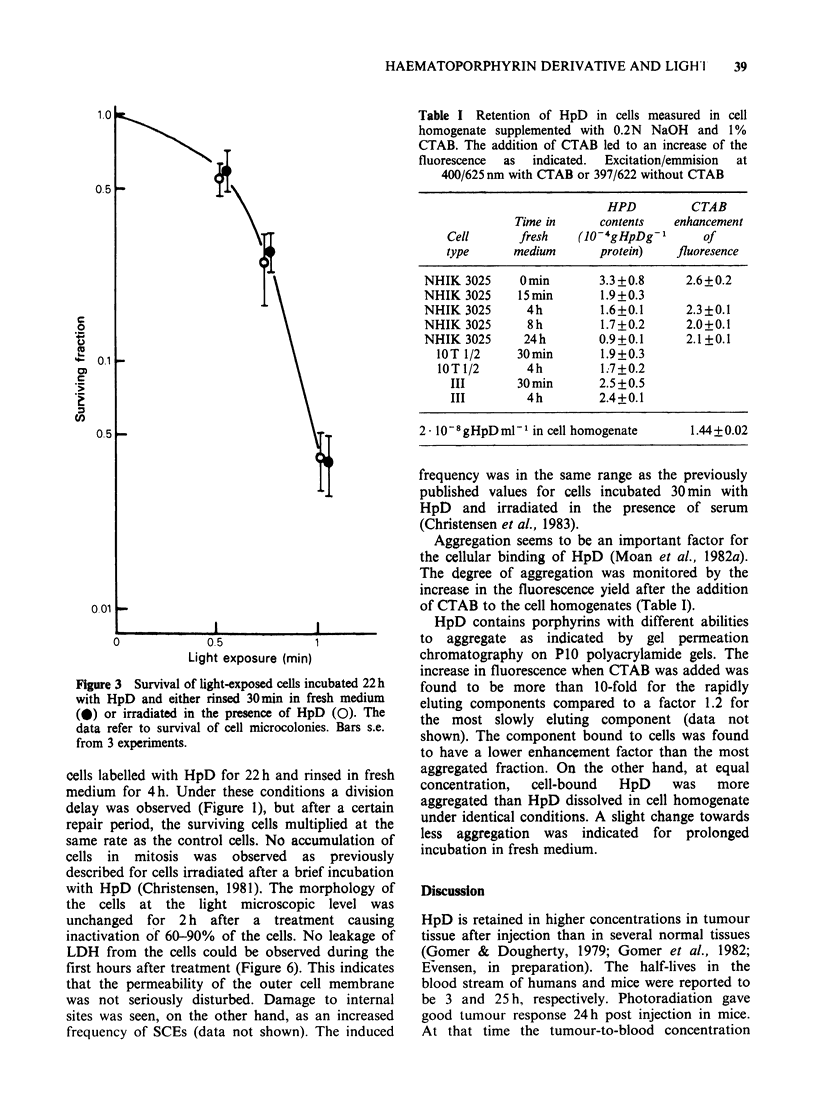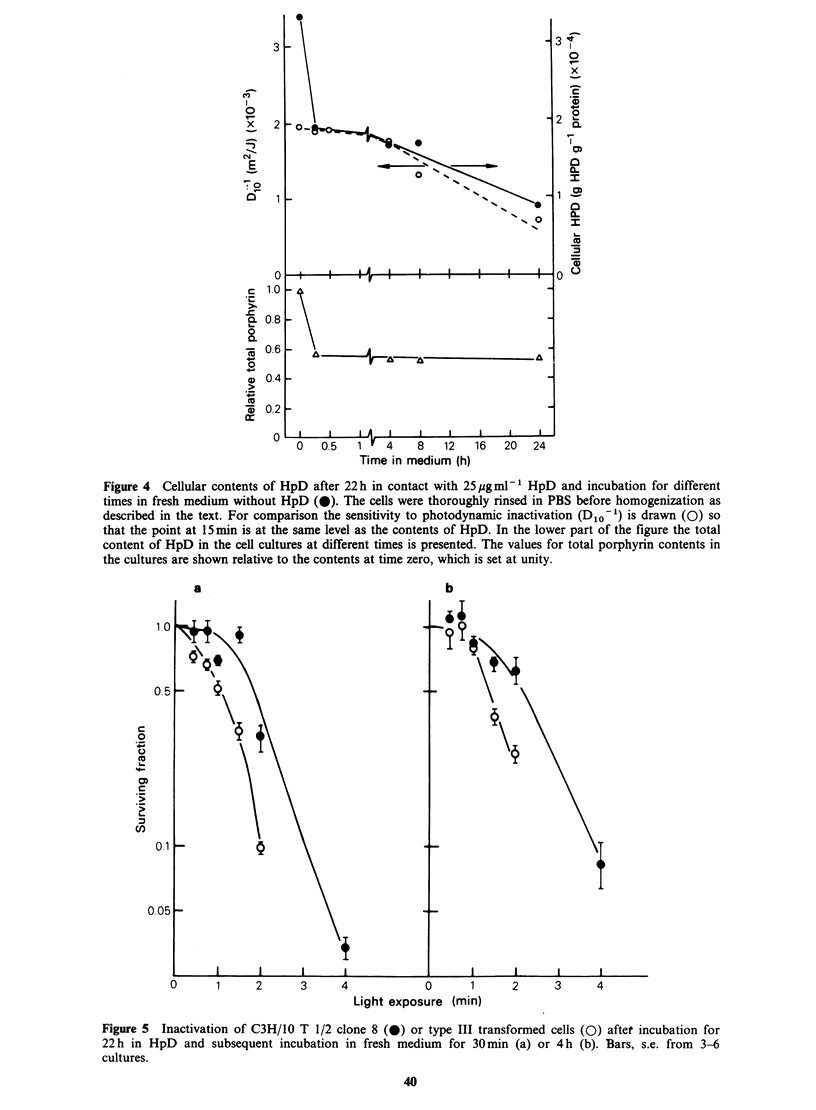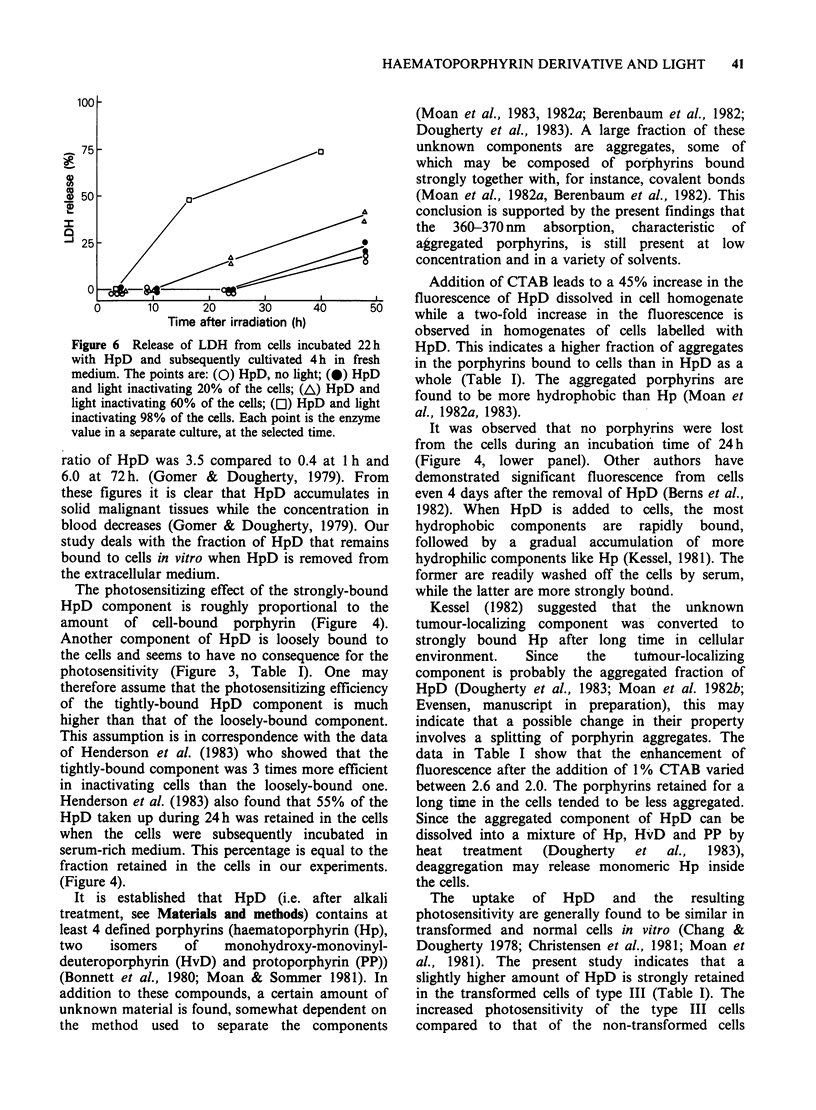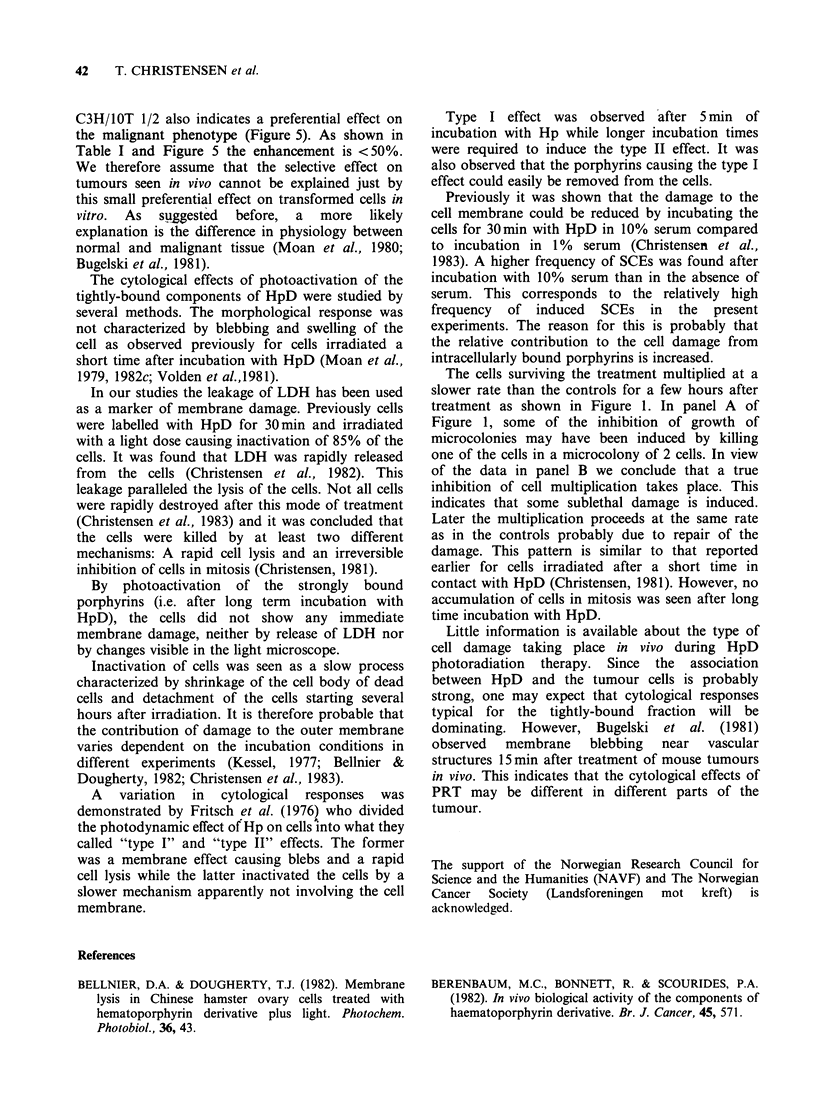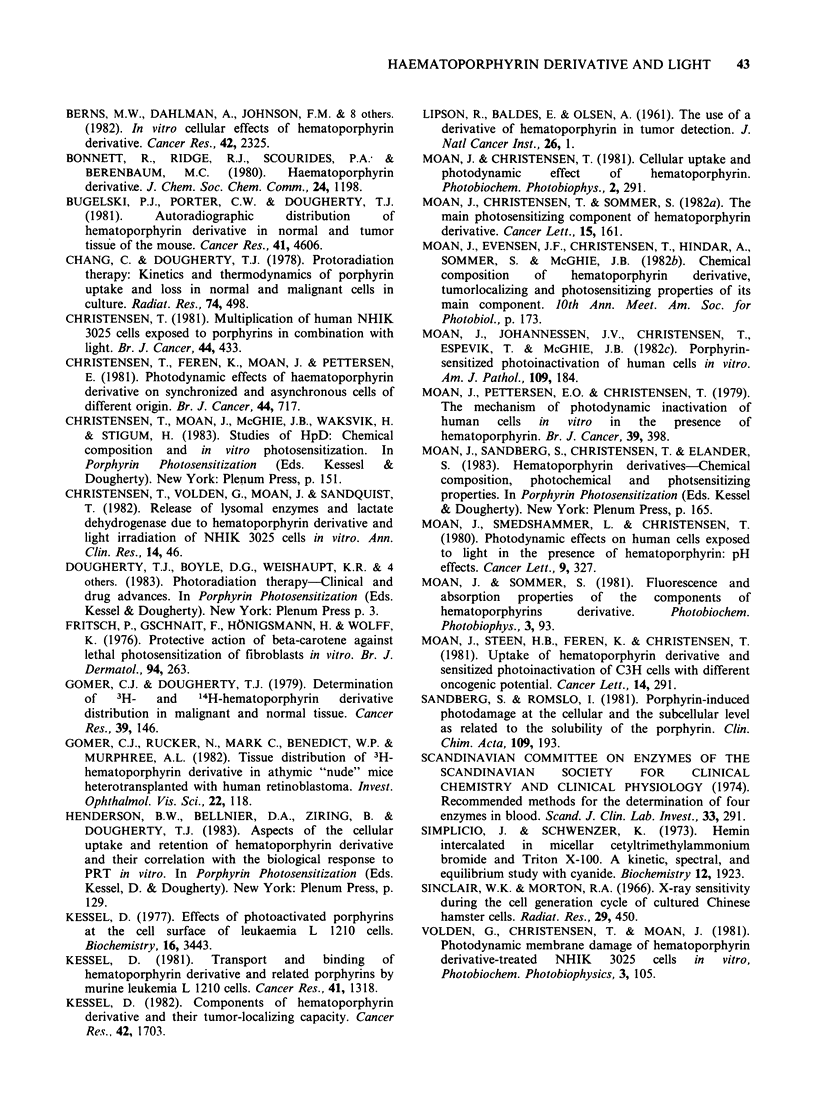# Retention and photodynamic effects of haematoporphyrin derivative in cells after prolonged cultivation in the presence of porphyrin.

**DOI:** 10.1038/bjc.1983.154

**Published:** 1983-07

**Authors:** T. Christensen, T. Sandquist, K. Feren, H. Waksvik, J. Moan

## Abstract

Photoradiation therapy of cancer in the presence of haematoporphyrin derivative is based on a retention of porphyrin in malignant tissue. After long term incubation of NHIK 3025 cells in the presence of 25 microgram ml-1 haematoporphyrin derivative, one fraction is easily removed from the cells by washing with a serum-rich medium. Another fraction remains bound to the cells for a prolonged time. The former does not contribute to the photosensitivity of the cells while the latter, the tightly-bound component, results in a photosensitivity proportional to the cellular contents of porphyrin. Transformed cells are shown to be slightly more sensitive and to retain 25-50% more haematoporphyrin derivative than non-transformed cells. Cytological effects of light absorbed by the tightly-bound component have been studied. The growth of treated cells is similar to that of control cells after a dose-dependent post irradiation lag period. A relatively slow leakage of lactate dehydrogenase (LDH) out of the cells takes place after treatment. The treatment induces a significant increase in the frequency of sister chromatid exchanges (SCE). We conclude that photoactivation of the tightly-bound fraction of haematoporphyrin derivative induces less damage to the outer cell membrane and probably more intracellular damage than irradiation of cells after a short period in contact with the derivative.


*Br. J. Cancer* (1983), **48**, 35–43

### Retention and photodynamic effects of haematoporphyrin derivative in cells after prolonged cultivation in the presence of porphyrin

T. Christensen[1], T. Sandquist[1], K. Feren[1,3], H. Waksvik[2] & J. Moan[1]

*Departments of [1]Biophysics, [2]Genetics and [3]Pathology, Norsk Hydro's Institute for Cancer Research, Montebello, Oslo 3, Norway*

**Summary** Photoradiation therapy of cancer in the presence of haematoporphyrin derivative is based on a retention of porphyrin in malignant tissue. After long term incubation of NHIK 3025 cells in the presence of $25\,\mu g\,ml^{-1}$ haematoporphyrin derivative, one fraction is easily removed from the cells by washing with a serum-rich medium. Another fraction remains bound to the cells for a prolonged time. The former does not contribute to the photosensitivity of the cells while the latter, the tightly-bound component, results in a photosensitivity proportional to the cellular contents of porphyrin. Transformed cells are shown to be slightly more sensitive and to retain 25–50% more haematoporphyrin derivative than non-transformed cells. Cytological effects of light absorbed by the tightly-bound component have been studied. The growth of treated cells is similar to that of control cells after a dose-dependent post irradiation lag period. A relatively slow leakage of lactate dehydrogenase (LDH) out of the cells takes place after treatment. The treatment induces a significant increase in the frequency of sister chromatid exchanges (SCE). We conclude that photoactivation of the tightly-bound fraction of haematoporphyrin derivative induces less damage to the outer cell membrane and probably more intracellular damage than irradiation of cells after a short period in contact with the derivative.

A gradual development in the understanding of the basic mechanisms behind photoradiation therapy (PRT) in the presence of haematoporphyrin derivative (HpD) has taken place during the last 5 years. An essential contribution to this progress has been the development of systems for chemical analysis of the HpD (Bonnett *et al.*, 1980, Moan *et al.*, 1982*a*; Kessel, 1982; Dougherty *et al.*, 1983). This has resulted in an improved drug, the purified porphyrin aggregates normally constituting ~40% of HpD (Dougherty *et al.*, 1983).

Cellular effects of photoactivated porphyrins have been shown to be different for different porphyrins (Sandberg & Romslo, 1981). In studies aimed to be relevant for the clinical use of PRT, cells should be exposed to the same porphyrin components and under similar conditions as *in vivo*. The precise *in vivo* conditions are as yet unknown. When used in PRT, the porphyrin is injected i.v. 2–7 days prior to photoradiation, in order to obtain a specific effect on the tumour (Dougherty *et al.*, 1983). The half life of HpD in the bloodstream is ~25 h (Gomer & Dougherty, 1979). The porphyrins must therefore in some way be retained in tumour tissue for a period of at least a few days. At the cellular level Kessel (1981) observed that HpD contains fractions able to bind strongly to cellular components. He also found differences between hydrophobic and hydrophilic components in their ability to be retained in cells. Generally the hydrophobic components were rapidly, but not strongly, bound, while some of the hydrophilic components were slowly taken up, bound tightly and retained in the cells.

Our group has previously reported that porphyrins are more strongly retained after prolonged incubation with HpD (Moan & Christensen, 1981). Furthermore, redistribution of porphyrins takes place within the cells causing changes in the photodynamic efficiency of the components (Kessel, 1981).

This paper describes the photodynamic properties of the components of HpD which are strongly bound to cells. The study shows a difference between cells with malignant and normal phenotypes *in vitro*. It is shown that the cellular effects of light irradiation of cells incubated with HpD for a prolonged time are different from the effects on the cells after short time incubation with HpD.

### Materials and methods

### *Cell cultivation*

Cells from the established line NHIK 3025 were grown in Minimal Essential Medium (MEM)

Correspondence: T. Christensen.

Received 17 January 1983; accepted 5 April 1983.

© The Macmillan Press Ltd., 1983

supplied with 10% newborn calf serum and antibiotics (Gibco, Scotland). The cells were subcultured twice a week and were thus in almost continuous logarithmic growth.

Cells from the line C3H/10T 1/2 clone 8 and DMBA transformed counterparts were routinely grown in Eagle's Basal medium with 10% foetal calf serum (Gibco). In this study cells from the parent line C3H/10T 1/2 clone 8 and type III transformed cells, both at passages 20–23, were compared. Before use in the experiments these cells were subcultured twice after storage in liquid $N_2$. Details of the cultivation of these cells have been published previously (Christensen *et al.*, 1981).

*Chemicals*

HpD was made according to the procedure suggested by S. Schwartz (Lipson *et al.*, 1961) in the following way: Haematoporphyrin dihydrochloride (Koch-Light) was treated with actetic acid and sulphuric acid and precipitated by addition of sodium acetate. The resulting product was dissolved to a concentration of $6.25\,mg\,ml^{-1}$ and stirred for 1 h at room temperature in 0.1N NaOH. Such treatment leads to hydrolysis of the acetates and the resulting mixture of porphyrins is termed HpD in accordance with Dougherty *et al.* (1983).

The biological activity of this HpD in a mouse transplantable tumour system has been tested. The tests have shown accumulation in mouse carcinomas as well as destruction of tumours upon irradiation with red light similar to the results of Gomer & Dougherty (1979) (Evensen, in preparation).

HpD was separated on a P10 polyacrylamide gel column (Bio-Rad) by eluting with PBS. This has been shown to result in fractions with different states of aggregation (Dougherty *et al.*, 1983).

*Irradiation*

The cells were inoculated in $25\,cm^2$ tissue culture flasks, either 2–3 h (NHIK 3025) or 20 h (C3H 10T 1/2 and type III) before the addition of $25\,\mu g\,ml^{-1}$ HpD in MEM with 10% newborn calf serum. In this period the cells were kept in the dark at 37°C. Twenty-two hours after the addition of HpD, the experiment was continued either by irradiation or by removal of the porphyrins. The cells were rinsed once in MEM containing 10% newborn calf serum. Fresh medium without HpD was added and the cells were left for variable periods before being subjected to light. The irradiation was performed with two Osram blacklight tubes irradiating the flasks from below (340–380 nm). At the position of the cells the light intensity was $11\,W\,m^{-2}$ as measured with a calibrated thermopile (YSI model 65 A).

*Sister chromatid exchanges*

NHIK 3025 cells ($5\times10^5$) were treated as above and SCEs were scored by differential staining of the chromatids according to the method described previously (Christensen *et al.*, 1983).

*HpD uptake*

Cells were cultivated as above except that they were inoculated in 60 mm Falcon petri dishes. Five ml of $25\,\mu g\,ml^{-1}$ HpD in MEM was added. This amount of HpD was large compared with that taken up in the cells. Thus, the HpD concentration was constant during the whole incubation period.

Labelling with HpD and rinsing with fresh medium was done by an exactly similar procedure to that described above. At selected times after removal of HpD, the dishes were washed 3 times in ice cold PBS and the cells were removed from the dishes with a Costar cell scraper. The cells were suspended in PBS, frozen and thawed once and sonicated. The fluorescence spectra were recorded with a Hitachi Perkin–Elmer spectrofluorimeter (MPF-2A) after addition of NaOH to 0.2N and CTAB (Cetyltrimethylammonium-bromide) to 1%. The contents of HpD in the cell homogentates was found by comparison with fluorescence from known amounts of HpD dissolved in homogenates in 0.2N NaOH and 1% CTAB of cells not labelled with HpD. The porphyrin contents were calculated per unit protein by running a protein assay (Bio-Rad) on each sample.

In order to use fluorescence as a quantitative measure for HpD uptake, it is important that the different components give the same yield of fluorescence. This was controlled by the following experiments. The components taken up by cells are probably partly aggregated and aggregates have a lower yield of fluorescence than monomers (Moan & Sommer, 1981). Therefore 1% CTAB was added in order to dissolve the porphyrin aggregates (Simplicio & Schwenzer 1973). The absorption at 360–370 nm is a parameter indicating the relative amount of porphyrin aggregates in a HpD solution (Moan & Sommer, 1981). The absorption spectrum of $10^{-6}\,g\,ml^{-1}$ HpD showed a shoulder at 360–370 nm when the porphyrin was dissolved in methanol/water, tetrahydrofurane, 1N NaOH or 4N HCl. The shoulder at 360–370 nm in the absorption spectrum of HpD in 1% CTAB was small compared with the other solvents mentioned. This indicates that most of the aggregates were dissolved. Similar results were obtained by Sommer (personal communication). He also found that the quantum yields of fluorescence from the components of HpD separated by HPLC were similar when the components were dissolved in CTAB micelles. Background fluorescence was assayed by incubating dishes without cells with HpD in cell culture medium exactly as described above. The dishes not containing cells were rinsed in PBS, and their bottoms were scraped with a cell scraper. This led to detachment of some porphyrins, probably bound to proteins attaching to the bottoms of the dishes. It was essential to control the background by doing this with replicate dishes at each data point because the background fluorescence tended to vary with incubation conditions even when no cells were present.

*Release of LDH*

After irradiation of $5 \times 10^5$ cells per flask previously labelled with HpD and rinsed for 4 h in fresh medium as described above, samples were collected from the medium at regular intervals. The contents of LDH were measured according to a standard method (Scandinavian Committee on Enzymes, 1974) by the Department of Clinical Chemistry at The Norwegian Radium Hospital.

*Cell multiplication*

Two methods were used to score cell multiplication after treatment. Microcolonies were exposed to HpD and light and the number of cells per colony was counted at selected times after treatment. The mean cell number per colony in 100 colonies was determined, and a new flask was used for each data point to avoid unwanted effects of light exposure from the microscope. The other method was based on the delineation of a fixed field containing ~100 cells in the bottom of each flask. One flask was used per light dose throughout and repeated counts of the cells in each field were performed in a dark room kept at 37°C. To avoid light exposure of the cells in the wavelength regions absorbed by HpD the light from the microscope lamp was filtered through 5 mm of a 5 mg $ml^{-1}$ HpD solution. A map showing the position of each cell within the field was drawn in order to control cell migration and cell death. Only cells that were present within the field during the whole experiment and their progeny were taken into account in the calculations of the cell number. Thus, cells that were killed by the treatment did not influence the results.

*Cell inactivation*

In order to score cell survival, NHIK 3025 cells were incubated for 10 days. At that time the resulting cell colonies were stained and counted. Survival curves were constructed. The sensitivity of the cells was expressed as $1/D_{10}$ where $D_{10}$ is the light dose reducing the surviving fraction from 1 to 0.1. During the incubation with HpD, some of the cells divided. Therefore, some of the cells were exposed to light while present in microcolonies of $\geq 2$ cells. The mean number of cells per colony during irradiation was determined, and the formula suggested by Sinclair & Morton (1966) was used to find the single cell survival. The C3H cells were incubated for 2 days after irradiation, trypsinized and counted as described previously (Christensen *et al.*, 1981).

### Results

The NHIK 3025 cells were in continuous exponential growth while exposed to HpD and subsequently to fresh growth medium (Figure 1). The plating efficiency of the cells was the same (70–90%) whether they were cultivated in the presence or absence of HpD. Light alone was neither toxic nor inhibitory of the multiplication of the cells.

In Figure 2 the sensitivity of NHIK 3025 cells is shown to decrease as a function of time after removal of HpD and addition of fresh medium. This decrease was gradual and the influence of a 30 min rinse in fresh medium was minimal with regard to photosensitivity (Figure 3). Surprisingly, a significant amount of porphyrin was removed from the cells by rinsing in fresh medium (Table I). This may indicate that a certain amount of the porphyrins bound to the cells after 22 h of incubation in 25 $\mu g\,ml^{-1}$ HpD did not have any consequence for the photosensitivity of the cells.

In Figure 4 the amount of porphyrin per protein unit in the cell culture is shown to decrease although the total contents of porphyrin in each culture are constant between 15 min and 24 h after the addition of fresh medium. A dilution of the porphyrin takes place because of cell division (Figure 1). No net transport of porphyrin out of the cells can be observed except for the decrease in cellular HpD content during the first minutes in fresh medium which is probably due to the binding of porphyrins to serum proteins (Moan *et al.*, 1979).

The gradual decrease in the porphyrin amount per cell between 15 min and 24 h led to a similar decrease in sensitivity (Figure 4).

A similar pattern was found when C3H/10T 1/2 cells and their transformed counterparts, type III, were tested (Table I, Figure 5). Transformed cells seemed to retain slightly more HpD and to be more sensitive than the non-transformed cells. The differences in retention and sensitivity were, however, small (25–50%).

Cytological effects of the strongly bound components of HpD were studied by irradiating

**Figure 1** Cell multiplication after 22 h in HpD and 4 h in fresh medium preceding exposure to light. (□) No HpD; (○) HpD, no light; (●) HpD and light inactivating 20% of the cells; (△) HpD and light inactivating 60% of the cells. In panel (a) the mean cell number per colony (±s.e.) in 100 microcolonies was scored and in panel (b) the increase in cell number within a fixed field was counted. In both panels the cell number shortly after inoculation was determined and the increase in cell number is given relative to the number of cells at inoculation.

**Figure 2** Survival of light-exposed cells incubated 22 h with HpD and subsequently 15 min (●) 4 h (○) 8 h (▲) or 24 h (△) in fresh medium with 10% serum before irradiation. In panel (a) the survival of microcolonies is shown. The data in panel (a) have been used to calculate the single cell survival shown in panel (b). Bars, s.e. from at least 3 experiments.

**Figure 3** Survival of light-exposed cells incubated 22 h with HpD and either rinsed 30 min in fresh medium (●) or irradiated in the presence of HpD (○). The data refer to survival of cell microcolonies. Bars s.e. from 3 experiments.

cells labelled with HpD for 22 h and rinsed in fresh medium for 4 h. Under these conditions a division delay was observed (Figure 1), but after a certain repair period, the surviving cells multiplied at the same rate as the control cells. No accumulation of cells in mitosis was observed as previously described for cells irradiated after a brief incubation with HpD (Christensen, 1981). The morphology of the cells at the light microscopic level was unchanged for 2 h after a treatment causing inactivation of 60–90% of the cells. No leakage of LDH from the cells could be observed during the first hours after treatment (Figure 6). This indicates that the permeability of the outer cell membrane was not seriously disturbed. Damage to internal sites was seen, on the other hand, as an increased frequency of SCEs (data not shown). The induced

**Table I** Retention of HpD in cells measured in cell homogenate supplemented with 0.2N NaOH and 1% CTAB. The addition of CTAB led to an increase of the fluorescence as indicated. Excitation/emmision at 400/625 nm with CTAB or 397/622 without CTAB

| *Cell type* | *Time in fresh medium* | *HPD contents ($10^{-4}$ g HpD g$^{-1}$ protein)* | *CTAB enhancement of fluoresence* |
|---|---|---|---|
| NHIK 3025 | 0 min | 3.3±0.8 | 2.6±0.2 |
| NHIK 3025 | 15 min | 1.9±0.3 | |
| NHIK 3025 | 4 h | 1.6±0.1 | 2.3±0.1 |
| NHIK 3025 | 8 h | 1.7±0.2 | 2.0±0.1 |
| NHIK 3025 | 24 h | 0.9±0.1 | 2.1±0.1 |
| 10T 1/2 | 30 min | 1.9±0.3 | |
| 10T1/2 | 4 h | 1.7±0.2 | |
| III | 30 min | 2.5±0.5 | |
| III | 4 h | 2.4±0.1 | |
| $2 \cdot 10^{-8}$ gHpD ml$^{-1}$ in cell homogenate | | | 1.44±0.02 |

frequency was in the same range as the previously published values for cells incubated 30 min with HpD and irradiated in the presence of serum (Christensen *et al.*, 1983).

Aggregation seems to be an important factor for the cellular binding of HpD (Moan *et al.*, 1982*a*). The degree of aggregation was monitored by the increase in the fluorescence yield after the addition of CTAB to the cell homogenates (Table I).

HpD contains porphyrins with different abilities to aggregate as indicated by gel permeation chromatography on P10 polyacrylamide gels. The increase in fluorescence when CTAB was added was found to be more than 10-fold for the rapidly eluting components compared to a factor 1.2 for the most slowly eluting component (data not shown). The component bound to cells was found to have a lower enhancement factor than the most aggregated fraction. On the other hand, at equal concentration, cell-bound HpD was more aggregated than HpD dissolved in cell homogenate under identical conditions. A slight change towards less aggregation was indicated for prolonged incubation in fresh medium.

### Discussion

HpD is retained in higher concentrations in tumour tissue after injection than in several normal tissues (Gomer & Dougherty, 1979; Gomer *et al.*, 1982; Evensen, in preparation). The half-lives in the blood stream of humans and mice were reported to be 3 and 25 h, respectively. Photoradiation gave good tumour response 24 h post injection in mice. At that time the tumour-to-blood concentration

**Figure 4** Cellular contents of HpD after 22 h in contact with 25 $\mu$g ml$^{-1}$ HpD and incubation for different times in fresh medium without HpD (●). The cells were thoroughly rinsed in PBS before homogenization as described in the text. For comparison the sensitivity to photodynamic inactivation ($D_{10}^{-1}$) is drawn (○) so that the point at 15 min is at the same level as the contents of HpD. In the lower part of the figure the total content of HpD in the cell cultures at different times is presented. The values for total porphyrin contents in the cultures are shown relative to the contents at time zero, which is set at unity.

**Figure 5** Inactivation of C3H/10 T 1/2 clone 8 (●) or type III transformed cells (○) after incubation for 22 h in HpD and subsequent incubation in fresh medium for 30 min (a) or 4 h (b). Bars, s.e. from 3–6 cultures.

**Figure 6** Release of LDH from cells incubated 22 h with HpD and subsequently cultivated 4 h in fresh medium. The points are: (○) HpD, no light; (●) HpD and light inactivating 20% of the cells; (△) HpD and light inactivating 60% of the cells; (□) HpD and light inactivating 98% of the cells. Each point is the enzyme value in a separate culture, at the selected time.

ratio of HpD was 3.5 compared to 0.4 at 1 h and 6.0 at 72 h. (Gomer & Dougherty, 1979). From these figures it is clear that HpD accumulates in solid malignant tissues while the concentration in blood decreases (Gomer & Dougherty, 1979). Our study deals with the fraction of HpD that remains bound to cells *in vitro* when HpD is removed from the extracellular medium.

The photosensitizing effect of the strongly-bound HpD component is roughly proportional to the amount of cell-bound porphyrin (Figure 4). Another component of HpD is loosely bound to the cells and seems to have no consequence for the photosensitivity (Figure 3, Table I). One may therefore assume that the photosensitizing efficiency of the tightly-bound HpD component is much higher than that of the loosely-bound component. This assumption is in correspondence with the data of Henderson *et al.* (1983) who showed that the tightly-bound component was 3 times more efficient in inactivating cells than the loosely-bound one. Henderson *et al.* (1983) also found that 55% of the HpD taken up during 24 h was retained in the cells when the cells were subsequently incubated in serum-rich medium. This percentage is equal to the fraction retained in the cells in our experiments. (Figure 4).

It is established that HpD (i.e. after alkali treatment, see **Materials and methods**) contains at least 4 defined porphyrins (haematoporphyrin (Hp), two isomers of monohydroxy-monovinyl-deuteroporphyrin (HvD) and protoporphyrin (PP)) (Bonnett *et al.*, 1980; Moan & Sommer 1981). In addition to these compounds, a certain amount of unknown material is found, somewhat dependent on the method used to separate the components (Moan *et al.*, 1983, 1982*a*; Berenbaum *et al.*, 1982; Dougherty *et al.*, 1983). A large fraction of these unknown components are aggregates, some of which may be composed of porphyrins bound strongly together with, for instance, covalent bonds (Moan *et al.*, 1982*a*, Berenbaum *et al.*, 1982). This conclusion is supported by the present findings that the 360–370 nm absorption, characteristic of aggregated porphyrins, is still present at low concentration and in a variety of solvents.

Addition of CTAB leads to a 45% increase in the fluorescence of HpD dissolved in cell homogenate while a two-fold increase in the fluorescence is observed in homogenates of cells labelled with HpD. This indicates a higher fraction of aggregates in the porphyrins bound to cells than in HpD as a whole (Table I). The aggregated porphyrins are found to be more hydrophobic than Hp (Moan *et al.*, 1982*a*, 1983).

It was observed that no porphyrins were lost from the cells during an incubation time of 24 h (Figure 4, lower panel). Other authors have demonstrated significant fluorescence from cells even 4 days after the removal of HpD (Berns *et al.*, 1982). When HpD is added to cells, the most hydrophobic components are rapidly bound, followed by a gradual accumulation of more hydrophilic components like Hp (Kessel, 1981). The former are readily washed off the cells by serum, while the latter are more strongly bound.

Kessel (1982) suggested that the unknown tumour-localizing component was converted to strongly bound Hp after long time in cellular environment. Since the tumour-localizing component is probably the aggregated fraction of HpD (Dougherty *et al.*, 1983; Moan *et al.* 1982*b*; Evensen, manuscript in preparation), this may indicate that a possible change in their property involves a splitting of porphyrin aggregates. The data in Table I show that the enhancement of fluorescence after the addition of 1% CTAB varied between 2.6 and 2.0. The porphyrins retained for a long time in the cells tended to be less aggregated. Since the aggregated component of HpD can be dissolved into a mixture of Hp, HvD and PP by heat treatment (Dougherty *et al.*, 1983), deaggregation may release monomeric Hp inside the cells.

The uptake of HpD and the resulting photosensitivity are generally found to be similar in transformed and normal cells *in vitro* (Chang & Dougherty 1978; Christensen *et al.*, 1981; Moan *et al.*, 1981). The present study indicates that a slightly higher amount of HpD is strongly retained in the transformed cells of type III (Table I). The increased photosensitivity of the type III cells compared to that of the non-transformed cells

C3H/10T 1/2 also indicates a preferential effect on the malignant phenotype (Figure 5). As shown in Table I and Figure 5 the enhancement is $<50\%$. We therefore assume that the selective effect on tumours seen *in vivo* cannot be explained just by this small preferential effect on transformed cells *in vitro*. As suggested before, a more likely explanation is the difference in physiology between normal and malignant tissue (Moan *et al.*, 1980; Bugelski *et al.*, 1981).

The cytological effects of photoactivation of the tightly-bound components of HpD were studied by several methods. The morphological response was not characterized by blebbing and swelling of the cell as observed previously for cells irradiated a short time after incubation with HpD (Moan *et al.*, 1979, 1982*c*; Volden *et al.*,1981).

In our studies the leakage of LDH has been used as a marker of membrane damage. Previously cells were labelled with HpD for 30 min and irradiated with a light dose causing inactivation of 85% of the cells. It was found that LDH was rapidly released from the cells (Christensen *et al.*, 1982). This leakage paralleled the lysis of the cells. Not all cells were rapidly destroyed after this mode of treatment (Christensen *et al.*, 1983) and it was concluded that the cells were killed by at least two different mechanisms: A rapid cell lysis and an irreversible inhibition of cells in mitosis (Christensen, 1981).

By photoactivation of the strongly bound porphyrins (i.e. after long term incubation with HpD), the cells did not show any immediate membrane damage, neither by release of LDH nor by changes visible in the light microscope.

Inactivation of cells was seen as a slow process characterized by shrinkage of the cell body of dead cells and detachment of the cells starting several hours after irradiation. It is therefore probable that the contribution of damage to the outer membrane varies dependent on the incubation conditions in different experiments (Kessel, 1977; Bellnier & Dougherty, 1982; Christensen *et al.*, 1983).

A variation in cytological responses was demonstrated by Fritsch *et al.* (1976) who divided the photodynamic effect of Hp on cells into what they called "type I" and "type II" effects. The former was a membrane effect causing blebs and a rapid cell lysis while the latter inactivated the cells by a slower mechanism apparently not involving the cell membrane.

Type I effect was observed after 5 min of incubation with Hp while longer incubation times were required to induce the type II effect. It was also observed that the porphyrins causing the type I effect could easily be removed from the cells.

Previously it was shown that the damage to the cell membrane could be reduced by incubating the cells for 30 min with HpD in 10% serum compared to incubation in 1% serum (Christensen *et al.*, 1983). A higher frequency of SCEs was found after incubation with 10% serum than in the absence of serum. This corresponds to the relatively high frequency of induced SCEs in the present experiments. The reason for this is probably that the relative contribution to the cell damage from intracellularly bound porphyrins is increased.

The cells surviving the treatment multiplied at a slower rate than the controls for a few hours after treatment as shown in Figure 1. In panel A of Figure 1, some of the inhibition of growth of microcolonies may have been induced by killing one of the cells in a microcolony of 2 cells. In view of the data in panel B we conclude that a true inhibition of cell multiplication takes place. This indicates that some sublethal damage is induced. Later the multiplication proceeds at the same rate as in the controls probably due to repair of the damage. This pattern is similar to that reported earlier for cells irradiated after a short time in contact with HpD (Christensen, 1981). However, no accumulation of cells in mitosis was seen after long time incubation with HpD.

Little information is available about the type of cell damage taking place *in vivo* during HpD photoradiation therapy. Since the association between HpD and the tumour cells is probably strong, one may expect that cytological responses typical for the tightly-bound fraction will be dominating. However, Bugelski *et al.* (1981) observed membrane blebbing near vascular structures 15 min after treatment of mouse tumours *in vivo*. This indicates that the cytological effects of PRT may be different in different parts of the tumour.

The support of the Norwegian Research Council for Science and the Humanities (NAVF) and The Norwegian Cancer Society (Landsforeningen mot kreft) is acknowledged.

**References**

BELLNIER, D.A. & DOUGHERTY, T.J. (1982). Membrane lysis in Chinese hamster ovary cells treated with hematoporphyrin derivative plus light. *Photochem. Photobiol.*, **36**, 43.

BERENBAUM, M.C., BONNETT, R. & SCOURIDES, P.A. (1982). *In vivo* biological activity of the components of haematoporphyrin derivative. *Br. J. Cancer*, **45**, 571.

BERNS, M.W., DAHLMAN, A., JOHNSON, F.M. & 8 others. (1982). *In vitro* cellular effects of hematoporphyrin derivative. *Cancer Res.*, **42**, 2325.

BONNETT, R., RIDGE, R.J., SCOURIDES, P.A. & BERENBAUM, M.C. (1980). Haematoporphyrin derivative. *J. Chem. Soc. Chem. Comm.*, **24**, 1198.

BUGELSKI, P.J., PORTER, C.W. & DOUGHERTY, T.J. (1981). Autoradiographic distribution of hematoporphyrin derivative in normal and tumor tissue of the mouse. *Cancer Res.*, **41**, 4606.

CHANG, C. & DOUGHERTY, T.J. (1978). Protoradiation therapy: Kinetics and thermodynamics of porphyrin uptake and loss in normal and malignant cells in culture. *Radiat. Res.*, **74**, 498.

CHRISTENSEN, T. (1981). Multiplication of human NHIK 3025 cells exposed to porphyrins in combination with light. *Br. J. Cancer*, **44**, 433.

CHRISTENSEN, T., FEREN, K., MOAN, J. & PETTERSEN, E. (1981). Photodynamic effects of haematoporphyrin derivative on synchronized and asynchronous cells of different origin. *Br. J. Cancer*, **44**, 717.

CHRISTENSEN, T., MOAN, J., McGHIE, J.B., WAKSVIK, H. & STIGUM, H. (1983). Studies of HpD: Chemical composition and *in vitro* photosensitization. In *Porphyrin Photosensitization* (Eds. Kessesl & Dougherty). New York: Plenum Press, p. 151.

CHRISTENSEN, T., VOLDEN, G., MOAN, J. & SANDQUIST, T. (1982). Release of lysomal enzymes and lactate dehydrogenase due to hematoporphyrin derivative and light irradiation of NHIK 3025 cells *in vitro*. *Ann. Clin. Res.*, **14**, 46.

DOUGHERTY, T.J., BOYLE, D.G., WEISHAUPT, K.R. & 4 others. (1983). Photoradiation therapy—Clinical and drug advances. In *Porphyrin Photosensitization* (Eds. Kessel & Dougherty). New York: Plenum Press p. 3.

FRITSCH, P., GSCHNAIT, F., HÖNIGSMANN, H. & WOLFF, K. (1976). Protective action of beta-carotene against lethal photosensitization of fibroblasts *in vitro*. *Br. J. Dermatol.*, **94**, 263.

GOMER, C.J. & DOUGHERTY, T.J. (1979). Determination of $^{3}$H- and $^{14}$H-hematoporphyrin derivative distribution in malignant and normal tissue. *Cancer Res.*, **39**, 146.

GOMER, C.J., RUCKER, N., MARK C., BENEDICT, W.P. & MURPHREE, A.L. (1982). Tissue distribution of $^{3}$H-hematoporphyrin derivative in athymic "nude" mice heterotransplanted with human retinoblastoma. *Invest. Ophthalmol. Vis. Sci.*, **22**, 118.

HENDERSON, B.W., BELLNIER, D.A., ZIRING, B. & DOUGHERTY, T.J. (1983). Aspects of the cellular uptake and retention of hematoporphyrin derivative and their correlation with the biological response to PRT *in vitro*. In *Porphyrin Photosensitization* (Eds. Kessel, D. & Dougherty). New York: Plenum Press, p. 129.

KESSEL, D. (1977). Effects of photoactivated porphyrins at the cell surface of leukaemia L 1210 cells. *Biochemistry*, **16**, 3443.

KESSEL, D. (1981). Transport and binding of hematoporphyrin derivative and related porphyrins by murine leukemia L 1210 cells. *Cancer Res.*, **41**, 1318.

KESSEL, D. (1982). Components of hematoporphyrin derivative and their tumor-localizing capacity. *Cancer Res.*, **42**, 1703.

LIPSON, R., BALDES, E. & OLSEN, A. (1961). The use of a derivative of hematoporphyrin in tumor detection. *J. Natl Cancer Inst.*, **26**, 1.

MOAN, J. & CHRISTENSEN, T. (1981). Cellular uptake and photodynamic effect of hematoporphyrin. *Photobiochem. Photobiophys.*, **2**, 291.

MOAN, J., CHRISTENSEN, T. & SOMMER, S. (1982*a*). The main photosensitizing component of hematoporphyrin derivative. *Cancer Lett.*, **15**, 161.

MOAN, J., EVENSEN, J.F., CHRISTENSEN, T., HINDAR, A., SOMMER, S. & McGHIE, J.B. (1982*b*). Chemical composition of hematoporphyrin derivative, tumorlocalizing and photosensitizing properties of its main component. *10th Ann. Meet. Am. Soc. for Photobiol.*, p. 173.

MOAN, J., JOHANNESSEN, J.V., CHRISTENSEN, T., ESPEVIK, T. & McGHIE, J.B. (1982*c*). Porphyrin-sensitized photoinactivation of human cells *in vitro*. *Am. J. Pathol.*, **109**, 184.

MOAN, J., PETTERSEN, E.O. & CHRISTENSEN, T. (1979). The mechanism of photodynamic inactivation of human cells *in vitro* in the presence of hematoporphyrin. *Br. J. Cancer*, **39**, 398.

MOAN, J., SANDBERG, S., CHRISTENSEN, T. & ELANDER, S. (1983). Hematoporphyrin derivatives—Chemical composition, photochemical and photsensitizing properties. In *Porphyrin Photosensitization* (Eds. Kessel & Dougherty). New York: Plenum Press, p. 165.

MOAN, J., SMEDSHAMMER, L. & CHRISTENSEN, T. (1980). Photodynamic effects on human cells exposed to light in the presence of hematoporphyrin: pH effects. *Cancer Lett.*, **9**, 327.

MOAN, J. & SOMMER, S. (1981). Fluorescence and absorption properties of the components of hematoporphyrins derivative. *Photobiochem. Photobiophys.*, **3**, 93.

MOAN, J., STEEN, H.B., FEREN, K. & CHRISTENSEN, T. (1981). Uptake of hematoporphyrin derivative and sensitized photoinactivation of C3H cells with different oncogenic potential. *Cancer Lett.*, **14**, 291.

SANDBERG, S. & ROMSLO, I. (1981). Porphyrin-induced photodamage at the cellular and the subcellular level as related to the solubility of the porphyrin. *Clin. Chim. Acta*, **109**, 193.

SCANDINAVIAN COMMITTEE ON ENZYMES OF THE SCANDINAVIAN SOCIETY FOR CLINICAL CHEMISTRY AND CLINICAL PHYSIOLOGY (1974). Recommended methods for the determination of four enzymes in blood. *Scand. J. Clin. Lab. Invest.*, **33**, 291.

SIMPLICIO, J. & SCHWENZER, K. (1973). Hemin intercalated in micellar cetyltrimethylammonium bromide and Triton X-100. A kinetic, spectral, and equilibrium study with cyanide. *Biochemistry* **12**, 1923.

SINCLAIR, W.K. & MORTON, R.A. (1966). X-ray sensitivity during the cell generation cycle of cultured Chinese hamster cells. *Radiat. Res.*, **29**, 450.

VOLDEN, G., CHRISTENSEN, T. & MOAN, J. (1981). Photodynamic membrane damage of hematoporphyrin derivative-treated NHIK 3025 cells *in vitro*, *Photobiochem. Photobiophysics*, **3**, 105.